# The Doubly Conditioned Frequency Spectrum Does Not Distinguish between Ancient Population Structure and Hybridization

**DOI:** 10.1093/molbev/msu103

**Published:** 2014-03-13

**Authors:** Anders Eriksson, Andrea Manica

**Affiliations:** ^1^Evolutionary Ecology Group, Department of Zoology, University of Cambridge, Cambridge, United Kingdom; ^2^Integrative Systems Biology Laboratory, King Abdullah University of Science and Technology (KAUST), Thuwal 23955-6900, Kingdom of Saudi Arabia

**Keywords:** hybridization, Neandertal, demography, population structure

## Abstract

Distinguishing between hybridization and population structure in the ancestral species is a key challenge in our understanding of how permeable species boundaries are to gene flow. The doubly conditioned frequency spectrum (dcfs) has been argued to be a powerful metric to discriminate between these two explanations, and it was used to argue for hybridization between Neandertal and anatomically modern humans. The shape of the observed dcfs for these two species cannot be reproduced by a model that represents ancient population structure in Africa with two populations, while adding hybridization produces realistic shapes. In this letter, we show that this result is a consequence of the spatial coarseness of the demographic model and that a spatially structured stepping stone model can generate realistic dcfs without hybridization. This result highlights how inferences on hybridization between recently diverged species can be strongly affected by the choice of how population structure is represented in the underlying demographic model. We also conclude that the dcfs has limited power in distinguishing between the signals left by hybridization and ancient structure.

Hybridization between different species can play a major role in evolution, both by bringing novel adaptations into species as well as by acting as a barrier to their divergence (Seehausen 2004; Abbott et al. 2013). However, detecting hybridization from genetic data can be challenging, as it requires distinguishing actual gene flow after the species split from shared variation that was present in the ancestral species (Abbott et al. 2013; Smith and Kronforst 2013; Sousa and Hey 2013). This problem is particularly challenging when considering hybridization among recently diverged species, where past population structure in the ancestral species can leave genetic signatures that are almost identical to those left by hybridization (Green et al. 2010; Eriksson and Manica 2012; Lowery et al. 2013).

The challenges of distinguishing between actual hybridization and ancient population structure have been highlighted by the recent publication of Neandertal genomes (Green et al. 2010; Prüfer et al. 2013). The main finding coming out of the first analysis of the draft sequence of the Neandertal genome (Green et al. 2010) was that populations of anatomically modern humans (AMHs) differed in genetic similarity to Neandertal. Specifically, modern Europeans and Asians were significantly more genetically similar to this hominin than Africans (Green et al. 2010). Patterson’s *D* statistics (SOM 15 in Green et al. 2010) is arguably the best-known approach to quantify this pattern. This statistics is based on a panel of four individuals and focuses on biallelic sites where either the Eurasian or the African match the Neandertal (but not both) and where the Neandertal is different from the chimp. *D* is calculated as the fraction of such sites where the Eurasian genome matches the Neandertal minus the fraction where the African genome matches Neandertal. In a simple four-population model without hybridization, we expect Eurasian and African genomes to have the same probability of matching the Neandertal through incomplete lineage sorting, but hybridization between Neandertal and one of the modern human populations would give rise to an unbalance (Green et al. 2010). An analysis using Patterson’s *D* revealed that the observed values for Neandertal were more extreme than expected by chance and were taken as evidence for hybridization (Green et al. 2010). This test has been used in a number of other taxa, such as primates (Prüfer et al. 2012), flycatchers (Rheindt et al. 2013), and Heliconius butterflies (Martin et al. 2013). However, a problem in interpreting Patterson’s *D* is that ancestral population structure can produce patterns undistinguishable from hybridization (Durand et al. 2011). In the case of Neandertal, a spatially structured model with realistic demographic parameters can produce *D* values identical to the ones measured from real genomes, even in the absence of hybridization (Eriksson and Manica 2012).

In an attempt to increase the power to detect hybridization, Yang et al. (2012) focused on the frequency distribution of Neandertal alleles in Eurasian populations at biallelic loci where Neandertal differ from the chimpanzee reference genome and modern-day Africans have the chimp allele. These loci have been called “doubly conditioned,” as they need to have the same allele in a modern African genome and the chimp genome (first condition) but to differ between chimp and Neandertal genomes (second condition; see fig. 1*a* for a schematic representation). Such loci should, in principle, be enriched for mutations that occurred in the Neandertal line and subsequently entered the human line through hybridization, and their relative frequency (the doubly conditioned frequency spectrum, dcfs, shown in fig. 1*b*) should be an informative measure of the strength of hybridization. Yang et al. (2012) showed that a population genetics model that represents ancient structure in Africa with two populations (see fig. 2*a* and *b* for a graphical representation of this model) predicts a deficit of rare doubly conditioned alleles (e.g., of frequency one in the sample) compared to the frequencies estimated from real data. Adding hybridization to such a model, however, restored the appropriate shape of the doubly conditioned allele frequency spectrum. Thus, the dcfs seems to be an informative metric to distinguish between hybridization and ancient population structure, and this result has been taken as a confirmation of hybridization between Neandertal and AMHs (e.g., Sankararaman et al. 2012).

**Fig. 1.**
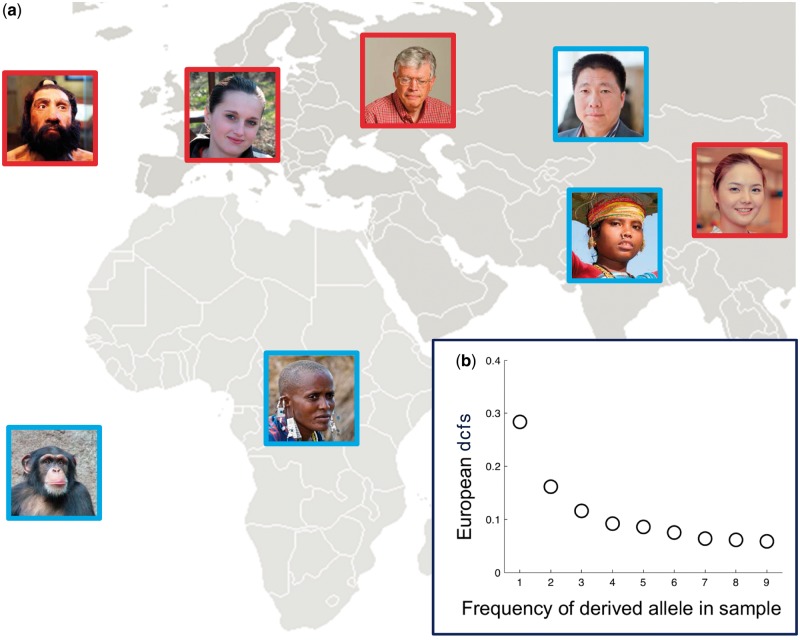
(*a*) A schematic representation on how the sample frequency of the Neandertal allele of a doubly conditioned locus is calculated. A locus is doubly conditioned if chimp and Neandertal have different alleles (shown in blue and red, respectively), and the ancestral chimp (blue) allele is found in Africa. The frequency of the Neandertal (red) allele is then estimated in the Eurasian panel: in this example, the frequency is 3. (*b*) Observed dcfs (the dcfs depicts the relative abundance of doubly conditioned loci with different derived allele frequencies), as estimated by Yang et al. (2012). Photographs from Wikipedia Commons, taken by T. Lersch, T. Evanson, W. Warby, Dyor, P. Neo, J. Montrasio, Y. Picq, and Fae.

**Fig. 2.**
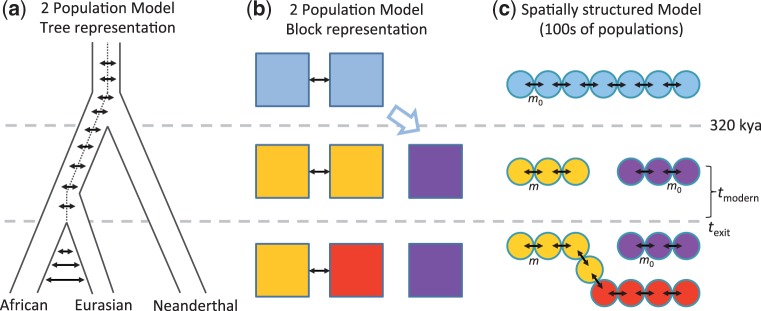
(*a*) Schematic representation of the “two-population model” in tree format. The ancestor of Neandertal and AMHs is structured into two populations. Neandertal splits from one of these two populations. The two populations keep exchanging migrants as they become AMHs, until that exchange decreases (but does not stop) when one population (the descendant of the parent population of Neandertal) leaves Africa to colonize Eurasia. (*b*) Block representation of the “two-population model,” where each block represents a population. (*c*) Schematic representation of the spatially structured model used in our analysis. The ancestor of Neandertal and AMHs is represented by a chain of interconnected populations with migration rate *m*_0_ (rather than just two as in the other model). The chain is separated into two when Neandertal speciates 320 kya, without any change in demographic parameters. Eventually, the African range becomes AMH at *t*_modern_, when its demography changes and the migration rate becomes *m*. At *t*_exit_, AMHs expand into Eurasia from the demes that were closest to the Neandertal range (note that the separation between Africa and Eurasia is generated by the range expansion and not by a change in migration rates, which stay at *m* throughout the AMH range).

However, it remains to be determined whether the dcfs can distinguish between hybridization and ancient structure when a spatially structured model with multiple populations is used instead of Yang et al.’s representation of ancient structure in the whole Africa continent with only two populations. Such spatially structured models better capture the global genetic clines in within-population genetic diversity observed in AMHs (Prugnolle et al. 2005; Ramachandran et al. 2005). Here we use the same spatially structured stepping stone model as previously presented in Eriksson and Manica (2012) to explore the properties of the dcfs with a fine-scale representation of ancient structure (fig. 2*c*, see supplementary material S1, Supplementary Material online, for details). Realistic demographic parameters were obtained by fitting the stepping stone to match worldwide patterns of spatial differentiation among modern populations and were further subsetted to focus on parameter combinations that predicted *D* between Africans and Europeans to be within 0.0020 U of the observed value 0.0457. This simple spatial model, which does not include any hybridization, predicts frequency spectra of doubly conditioned alleles (the dcfs) that are in line with observed values (gray lines and shaded ranges in fig. 3*a*), matching closely the empirical proportion of rare alleles (giving *R*^2 ^= 99.2% for the best fit). Some demographic parameter combinations give rise to a slight excess of very common alleles, but there are a large number of combinations that fit the observed dcfs almost perfectly (ten examples are shown as lines in fig. 3*a*, gray lines; see SOM for details). This spatially explicit model (which has eight free parameters) provides a fit that is comparable (*R*^2 ^= 99.2 vs. *R*^2 ^= 99.7%) to the admixture model in Yang et al (2012) (which has nine free parameters; blue line in fig. 3*a*). It is also considerably better than the best model fit for ancient population structure presented in Yang et al (2012), which has an *R*^2 ^= 93.7% (green line in fig. 3*a*). The large proportion of rare doubly conditioned alleles in our spatially structured model is a consequence of deep splits in gene genealogies, with old, relatively rare lineages being preserved by the fine-grained spatial structure in the model (fig. 3*b*). In other words, the presence of multiple (spatially structured) populations within Africa prevents lineages from coalescing too quickly, thereby allowing for a few European lineages to merge back with Neandertal before meeting any African lineage. In many cases, such lineages are only represented by one or two individuals, giving an excess of rare doubly conditioned loci.

**Fig. 3.**
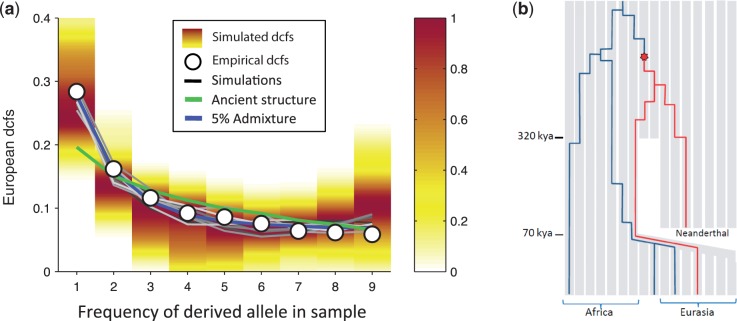
(*a*) Doubly conditioned frequency spectrum of Neandertal alleles in five Europeans. Circles represent the empirical dcfs observed in the data by Yang et al (2012), and the colored bars show the distribution predicted by our spatially structured model of ancient population structure. The shaded lines show predictions for ten different parameter combinations among the good fits. For comparison, we show Yang et al.’s best model of ancient population structure (green line) and admixture (blue line). In contrast to simple demographic models, our spatial model correctly captures the relative abundance of rare alleles (frequencies of 1 and 2 in the sample). (*b*) Schematic representation of how spatial structure occasionally prevents a Eurasian lineage (in red) from coalescing back with other Eurasian and Africa lineages (in blue), generating a rare doubly conditioned locus. The key mutation generating the Neandertal-like allele is highlighted by a red star. Note that time on the Neandertal branch was compressed to make room for the out-of-Africa expansion.

It is beyond the scope of this short letter to provide a formal test for alternative hybridization scenarios with Neandertal. Population structure affects a number of aspects of the similarities between Eurasians and Neandertal. For example, the degree of matching between ancient and derived SNPs in candidate regions for hybridization (SOM 17 in Green et al. [2010]) can be reproduced by a spatial model analogous to the one presented in this letter, without any hybridization (Eriksson and Manica 2012). A number of studies, including the first analyses of two new Neandertal genomes (Prüfer et al. 2013), provides an intricate picture of possible hybridization events among a number of hominins. Possibly, the clearest analysis pointing to hybridization is the dating of the Neandertal gene flow into modern humans based on linkage disequilibrium patterns (Sankararaman et al. 2012). However, such dates are based on the same demographic representation used in Yang et al. (2012). Thus, it will be interesting to see whether linkage disequilibrium patterns are affected by different spatial representations of population structure or not.

In general, the very different results obtained by a model that represents genetic structure in Africa with two populations (Yang et al. 2012) versus our spatially structured model highlight the importance of the coarseness at which space is described. When investigating hybridization, especially in the case of recently diverged species, metrics have been devised to focus the power of the analysis on the key signals that would be expected from hybridization. However, spatial structuring of populations can easily mimic such signals. No matter how sophisticated the metrics are, the properties of different demographic models should be explored, in particular how robust the analysis is to the spatial scale of demographic processes.

## Supplementary Material

Supplementary Data
