# Supplementary material for: The Doubly Conditioned Frequency Spectrum Does Not Distinguish between Ancient Population Structure and Hybridization
Source: Mol Biol Evol. 2014 Mar 13;31(6):1618–21. doi: 10.1093/molbev/msu103 (PMC4032131; doi:10.1093/molbev/msu103)
Supplement: Supplementary Data [file supp_31_6_1618__index.html]

The Doubly-Conditioned Frequency Spectrum does not distinguish between ancient population structure and hybridisation — The Doubly Conditioned Frequency Spectrum Does Not Distinguish between Ancient Population Structure and Hybridization — The Doubly Conditioned Frequency Spectrum Does Not Distinguish between Ancient Population Structure and Hybridization — Supplementary Data 

# The Doubly Conditioned Frequency Spectrum Does Not Distinguish between Ancient Population Structure and Hybridization

## Supplementary Data

files

**Files in this Data Supplement:**

- Supplementary Data - pdf file
