# Supplementary material for: The Doubly Conditioned Frequency Spectrum Does Not Distinguish between Ancient Population Structure and Hybridization
Source: Mol Biol Evol. 2014 Mar 13;31(6):1618–21. doi: 10.1093/molbev/msu103 (PMC4032131; doi:10.1093/molbev/msu103)
Supplement: Supplementary Data [file supp_msu103_DCFS_and_Neadnerthal_Supplementary_20140303.pdf]

Supplementary Material

Methods

The demographic model

We represent the joint history of anatomically modern humans and Neandertal with a spatially structured stepping stone model (Fig. 2c), using the same setup adopted by Eriksson and Manica (2012). Briefly, we consider a common ancestor of the two hominins which occupied a string of demes (separated by 100 kilometres) spanning Africa and Eurasia (130 demes in total). Each deme contained K_0 individuals and, at each generation (corresponding to 25 years), exchanged m_0K_0 migrants with its two adjacent neighbours. At 320 kya, the range was split into two areas, Africa (where anatomically modern humans evolved) and Eurasia (the home of Neandertal), by placing a barrier at deme 70 across which no migration was allowed (thus generating two separate strings of demes, one 70 demes long representing Africa, and one 60 demes long representing Eurasia).

At time t_{modern}, when anatomically modern humans were still confined to Africa, the demographic parameters were changed to modern values, with carrying capacity going from K_0 to K and migration rate from m_0 to m. At t_{exit}, the barrier preventing the exit out of Africa was removed, and modern humans were allowed to colonise Eurasia. This process occurred over a branch of the stepping stone model parallel to the Eurasian line already occupied by Neandertal (thus allowing the coexistence of the two hominins), but in this case extending 260 demes (to represent the full stretch from into the Americas), instead of the 60 demes used to represent the more limited Eurasian Neandertal range. The spread of AMHs into Eurasia occurred via sequential founder events, with new demes being colonised by cK new individuals. After colonisation, the population sizes grew by rK individuals per generation, until they reached K. Each pair of adjacent occupied demes exchanged N_{min} migrants per generation, where N_{min} represent the smaller of the two population sizes.

Parameterising the model

Since our aim is to explore the expected patterns of *dcfs* under a null scenario of population structure, it is crucial to choose demographic parameters that provide a realistic representation of present and past structure. We fitted our model to estimates of within and between population Time to Most Recent Common Ancestor (TMRCA) from the HGDP-CEPH panel (Cann et al. 2002), which includes over 1000 individuals from 51 populations across the globe. The HGDP-CEPH panel arguably provides the best overview of global genetic diversity in modern humans. Thus, we restrict our analysis to demographic parameters that are compatible with the genetic variation of modern humans.

TMRCAs were calculated from the mean square difference of repeat counts in di- and tri-nucleotide microsatellite markers (Eriksson and Manica 2011), genotyped in individuals from the HGDP-CEPH panel. Di-nucleotide markers where calibrated using the mutation rate of Dib et al. (1996), μ = 1.52 x 10^{-3} single-step mutations per 27 years (i.e. μ = 1.41 x 10^{-3} per generation). TMRCA of tri-nucleotide markers were scaled to match the average TMRCA of the di-nucleotide markers (Eriksson and Manica 2011).

The predicted TMRCA for a given parameter combination was calculated as follows: we first ran the demographic model described in the previous section, and then generated 100 gene genealogies for 10 individuals in each of the 51 populations corresponding to the HGDP-CEPH populations in our data [placed according to the deme corresponding to the distance from a location in sub-Saharan Africa, calculated using shortest distances on land as in Prugnolle et al. (2005)]. We then traced gene genealogies backwards in time, generation by generation, assuming diploid, random mating within each colonised deme, and with migration probabilities to neighbouring demes given by the demographic model.

We fitted our model in the Approximate Bayesian Computation (ABC) framework, using the ABC-GLM algorithm implemented in the ABCtoolbox software (Wegmann et al. 2010). We generated six summary statistics from the average TMRCA between continents. We treated Europe and Central

Asia as one continent (Eurasia), and East Asia as a separate continent. Because Oceania only has two populations (both in Papua New Guinea), we included these populations in the East Asian set. Our summary statistics are thus $T_{Africa,Eurasia}$, $T_{Africa,EastAsia}$, $T_{Africa,America}$, $T_{Eurasia,EastAsia}$, $T_{Eurasia,America}$, and $T_{America,America}$ (empirical values are 176.1 kya, 143.9 kya, 131.7 kya, and 105.7 kya, respectively).

We started by randomly sampling 2.2 million parameter values from the following ranges: $m \in [10^{-6}, 0.33]$, $c \in [10^{-4}, 0.33]$, $r \in [0.01, 1]$, $K \in [10, 10^5]$, $K_0 \in [10, 10^5]$, $m_0 \in [10^{-6}, 0.33]$, $t_{\text{modern}} \in [100, 200]$ (k years ago) and $t_{\text{exit}} \in [40, 80]$ (k years ago). All parameters (with the exception of t_{modern} and t_{exit}) were log-transformed to ensure an adequate exploration of the large ranges of possible values. We further imposed (through rejection sampling) the constraint $cK < K/2$ (cannot send out more colonists than individuals). Finally, we used ABC to estimate the likelihood of the 0.05% best-fitting parameter combinations [corresponding to 1115 parameter combinations; the same ones we used in Eriksson and Manica (2012)], and to generate parameter posterior distributions [see Fig. S2 in Eriksson and Manica (2012)]. This set was further subsetted to focus on parameter combinations that predicted *D* between Africans and Europeans to be within 0.0020 units of the observed value 0.0457.

Quantifying dcfs

We estimated the predicted *dcfs* for the best-fitting demographic parameter combinations, weighted by their likelihood as estimated by ABC. We should emphasize that we did not fit the model to the observed *dcfs*, but rather used realistic parameter combinations (based on the global distribution of genetic variation in modern populations) to predict *dcfs* under a null scenario without hybridisation. We attempted to match the sample design of Yang *et al.* (2012) as closely as possible. For each demographic parameter combination, we simulated 10 million unlinked SNPs in one African genome (placed in deme 10), five North European genomes (from deme 120), and the Neandertal genome (in deme 27 of their Eurasian range, corresponding to deme 97 of the AMHs longer chain). As in Eriksson and Manica (2012), we chose deme 27 as it represents the distance between the

Vindija cave in Croatia (the location of the material from which the Neandertal genome was extracted) and the point where the Neandertal branch separates from the human branch in the Middle East. Similarly, the other populations were chosen based on their distance from a putative sub-Saharan origin chosen as -12° latitude and 25° longitude based on Manica et al (Manica et al. 2007). We filtered the simulated SNPs for those compatible with the *dcfs* criteria, and then calculated the *dcfs* using the frequency of the Neandertal allele in the European genomes for each SNP. The ten best parameter combinations are shown in table S1, and the corresponding *dcfs* values are shown in table S2 (along with the empirical *dcfs* and the two models of Yang *et al.* shown in figure 3).

References

Cann HM, de Toma C, Cazes L, et al. 2002. A human genome diversity cell line panel. Science 296:261–262.

Dib C, Fauré S, Fizames C, et al. 1996. A comprehensive genetic map of the human genome based on 5,264 microsatellites. Nature 380:152–154.

Eriksson A, Manica A. 2011. Detecting and Removing Ascertainment Bias in Microsatellites from the HGDP-CEPH Panel. G3-Genes Genomes Genet. 1:479–488.

Eriksson A, Manica A. 2012. Effect of ancient population structure on the degree of polymorphism shared between modern human populations and ancient hominins. Proc. Natl. Acad. Sci. 109:13956–13960.

Manica A, Amos W, Balloux F, Hanihara T. 2007. The effect of ancient population bottlenecks on human phenotypic variation. Nature 448:346–348.

Prugnolle F, Manica A, Balloux F. 2005. Geography predicts neutral genetic diversity of human populations. Curr. Biol. 15:R159–60.

Wegmann D, Leuenberger C, Neuenschwander S, Excoffier L. 2010. ABCtoolbox: a versatile toolkit for approximate Bayesian computations. BMC Bioinformatics 11.

Yang MA, Malaspinas A-S, Durand EY, Slatkin M. 2012. Ancient structure in Africa unlikely to explain neanderthal and non-african genetic similarity. Mol. Biol. Evol. 29:2987–2995.

Table S1 Parameter values for the ten best fits to the empirical dcfs spectrum.

K	K_0	r	c	m	m_0	t_{exit}	t_{modern}
1021.4	21.939	0.671	0.1233	0.085233	0.11871	46	125.48
1509.6	24.34	0.14167	0.040777	0.13761	0.076934	69.6	109.7
1244.6	27.665	0.5085	0.098318	0.081384	0.079035	56.6	113.33
1062.8	14.363	0.39598	0.10295	0.063094	0.075023	60.425	135.95
971.79	12.568	0.44794	0.19421	0.096609	0.067592	56.85	124.38
875.23	27.686	0.21906	0.12269	0.15869	0.063905	75.025	105.18
21009	13.469	0.13024	0.0054827	0.048054	0.065207	62.175	126.9
1325.5	28.622	0.14714	0.13868	0.1152	0.10608	61.825	108.65
8452.9	22.188	0.81569	0.007889	0.12822	0.062028	70.575	109.85
12822	23.16	0.67825	0.0084355	0.091636	0.090976	57.9	115.78

Table S2 Dcfs values shown in figure 3.

Frequency	1	2	3	4	5	6	7	8	9
**Empirical**	0.284	0.162	0.116	0.092	0.086	0.075	0.064	0.062	0.059
**Yang *et al.* admixture**	0.278	0.159	0.111	0.089	0.079	0.074	0.072	0.069	0.070
**Yang *et al.* structure**	0.196	0.151	0.128	0.112	0.100	0.092	0.081	0.074	0.066
**This paper**	0.267	0.140	0.117	0.104	0.087	0.075	0.070	0.064	0.075
	0.284	0.158	0.102	0.074	0.074	0.081	0.081	0.075	0.070
	0.254	0.164	0.115	0.089	0.081	0.077	0.074	0.076	0.069
	0.272	0.137	0.113	0.098	0.102	0.091	0.063	0.061	0.063
	0.292	0.171	0.130	0.086	0.064	0.055	0.059	0.073	0.071
	0.271	0.161	0.112	0.087	0.071	0.066	0.067	0.073	0.091
	0.279	0.145	0.110	0.099	0.079	0.062	0.066	0.070	0.090
	0.263	0.149	0.117	0.101	0.078	0.063	0.067	0.076	0.086
	0.281	0.154	0.122	0.092	0.070	0.056	0.061	0.076	0.089
	0.292	0.144	0.099	0.078	0.075	0.075	0.079	0.080	0.077
